# Parathyroid hormone in relation to various vitamin D metabolites in adult females

**DOI:** 10.1097/MD.0000000000008071

**Published:** 2017-09-15

**Authors:** Nasser M. Al-Daghri, Sobhy Yakout, Ihtisham Bukhari, Malak N.K. Khattak, Yousef Al-Saleh, Naji Aljohani, Omar S. Al-Attas, Majed Alokail

**Affiliations:** a Biochemistry Department, College of Science, King Saud University; b College of Medicine, King Saud Bin Abdulaziz University for Health Sciences; c Obesity, Endocrine and Metabolism Center, King Fahad Medical City, Faculty of Medicine, King Saud bin Abdulaziz University for Health Sciences, Riyadh, Saudi Arabia.

**Keywords:** parathyroid hormone, vitamin D binding protein, vitamin D concentration

## Abstract

Vitamin D binding protein (DBP) and albumin are the important determinants of circulatory 25(OH)D in adults. Physiological function of vitamin D is particularly regulated by DBPs. Serum parathyroid hormone (PTH) is considered as the biological activity reader of circulating 25(OH)D. We therefore examined the relationships between serum total, free, and bioavailable 25(OH)D versus PTH in apparently healthy Saudi female adults.

A total of 350 apparently healthy Saudi female adults ([Mean ± standard deviation] age [years] 52.9 ± 9.2; body mass index [kg/m^2^] 32.9 ± 5.4) were included in this observational study. Anthropometrics was measured as well as fasting glucose, lipid profile, calcium and phosphorous using routine methods. Serum 25(OH)D was measured using an electrochemiluminescence immunoassay. Serum DBP was determined by ELISA. Free and bioavailable 25(OH)D were calculated by comparing concentrations of total 25(OH)D, DBP, and albumin.

Data revealed that circulating total 25(OH)D had weak but significant inverse association with DBP (R = −0.24; *P* < .01), and strong inverse associations with free 25(OH)D (R = −0.87; *P* < .001), albumin-bound 25(OH)D (R = −0.88; *P* < .001), and bioavailable 25(OH)D (R = −0.89; p < 0.001). None of the vitamin D metabolites, including 25(OH)D, correlated with serum PTH.

Various metabolites of 25(OH)D are not correlated with serum PTH in Saudi adult females. Bioavailable, albumin-bound and free 25(OH)D cannot be surrogate measures for vitamin D status, at least in this population.

## 1 Introduction

25-hydroxyvitamin D [25(OH)D] is the preferred metabolite that has been used to assess serum vitamin D levels. About 90% of the total 25(OH)D bound with vitamin D binding protein (DBP) for transport of target tissues, 10% makes loose bond with albumin and 0.1% is free circulating vitamin D.^[1]^ The binding affinity of DBP for vitamin D metabolites is > 1000-fold stronger than that of albumin (7 × 10^8^ vs 6 × 10^5^ M^−1^) for 25(OH)D and therefore, the albumin-bounded and free fractions together are considered bioavailable.^[2]^

In some cases, 25(OH)D may not be a reliable marker of vitamin D status because of its changeable nature in different conditions, like in free hormone levels, regardless of its total stored amount.^[3,4]^ DBP can be considered as a reservoir or modulator of the biological activity of vitamin D.^[5,6]^ The inhibitory effect of DBP on vitamin D activity may cause misunderstanding of the true vitamin D status. In some patients (liver cirrhosis) with low DBP, bioavailable vitamin D found is higher than the low total 25(OH)D.^[5]^ Also, increase in DBP can cause deficiency in levels of functional vitamin D and 25(OH)D.^[7]^

Hence, the relationship between vitamin D and other diseases varies considerably on the metabolite used for study (eg, total 25(OH)D or free vitamin D). Regardless, there are many benefits to measure free vitamin D, especially in the presence of abnormalities in DBP level. Therefore, the biological significance of vitamin D is very much dependent on the extent of free form rather than total vitamin D.^[6]^ Furthermore, it has been assumed that serum parathyroid hormone (PTH) can be a biological activity reader of 25(OH)D.^[8]^

To the best of our knowledge, few studies have been conducted to examine the relative relationships among total, free, and bioavailable 25(OH)D with PTH. The study is conducted to evaluate the indexing unit of vitamin D using free and bioavailable 25(OH)D. We therefore assessed the relationship between serum total, free, and bioavailable 25(OH)D and PTH with and without adjusting for DBP.

## 2 Methods

### 2.1 Subjects

In this cross-sectional study, a total of 350 healthy individuals (52.93 ± 9.19 years) were randomly recruited from primary public health care centers in Riyadh, Saudi Arabia. Information regarding age, height, weight, and socioeconomic status was provided by each participant on a structured questionnaire. Informed written consents were taken from all participants before collection of samples. Individuals, who had chronic diseases such as asthma and diabetes, were vitamin D deficient, or using vitamin D, calcium, and multivitamin supplementation, were excluded from study. The ethical review committee of the College of Science Research Center, King Saud University (KSU), Riyadh, Saudi Arabia has approved all the experimental and sampling procedures of this study. All the procedures are in accordance with the Helsinki Declaration 1964 and its later amendments. Predesigned and approved questionnaires were used to collect socio-demographics information and medical history.

### 2.2 Anthropometry and blood collection

All the individuals were asked to visit primary heath care centers for blood sampling and anthropometric measurements, including weight, height, waist and hip circumference, and mean diastolic and systolic blood pressure. Body mass index (BMI) was calculated by dividing weight (kilograms) by height (square meters). About 10 cm^3^ venous blood samples were collected from each individual and processed for separation of serum samples. Remaining blood samples and serum samples were transported to the Biomarkers Research Program (BRP) in King Saud University, Riyadh, Saudi Arabia in specialized containers for biochemical analyses and storage at −80°C.

### 2.3 Determination of serological parameters

Blood lipid profile, glucose, calcium, and phosphorous were measured using a chemical analyzer (Konelab, Espoo, Finland). Serum 25(OH)D was measured by Roche Elecsys modular analytics Cobas e411 using an electrochemiluminescence immunoassay (Roche Diagnostics, GmbH, Mannheim, Germany). Serum DBP was determined by ELISA (R&D Systems) with inter-assay CV (1.6–3.6%), and recovery of (98–103%). Free and bioavailable 25(OH)D were calculated by comparing with concentrations of total 25(OH)D, DBP, and albumin as per theory formulated by Powe et al^[9]^ as follow: 
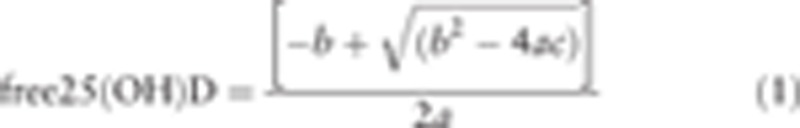
 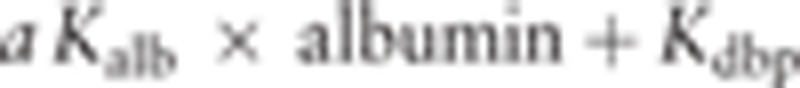 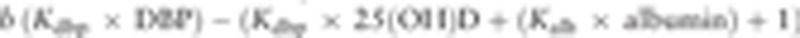


*K*_dpb_ affinity constant between 25(OH)D and DBP.

*K*_alb_ affinity constant between 25(OH)D and albumin. 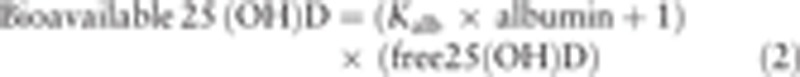


### 2.4 Statistical analyses

Data were analyzed using SPSS (version 22.0, Chicago, IL). Continuous data were presented as mean ± standard deviation (SD) for variables following Gaussian and non-Gaussian variables were presented in median (1st and 3rd) percentiles. Categorical data were presented as frequencies and percentages (%). All continuous variables were checked for normality using Kolmogorov-Smirnov test. Correlations between variables were done using Pearson correlation analysis. *P* value < .05 was considered statistically significant.

## 3 Results

### 3.1 Participant characteristics

A total of 350 Saudi healthy adults (average age 52.9 ± 9.2 years) were recruited. Vitamin D, DBP, and PTH were examined along with other biochemical, demographic, and anthropometric parameters. The overall median (interquartile range) for the serum concentrations of 25(OH)D, DBP, PTH, phosphorous, calcium, and albumin were 46.2 nmol/L, 72.8 ng/mL, 8.7 pg/mL, 1.1 ± 0.3 mmol/L, 2.3 ± 0.2, and 39.6 ± 4.3 mmol/L, respectively. The prevalence of vitamin D deficiency and insufficiency were high, 20.6% (n = 71) and 30.7% (n = 106), respectively; 27.5% of the individuals had circulating 25(OH)D levels higher than 75 nmol/L (Table 1).

**Table 1:**
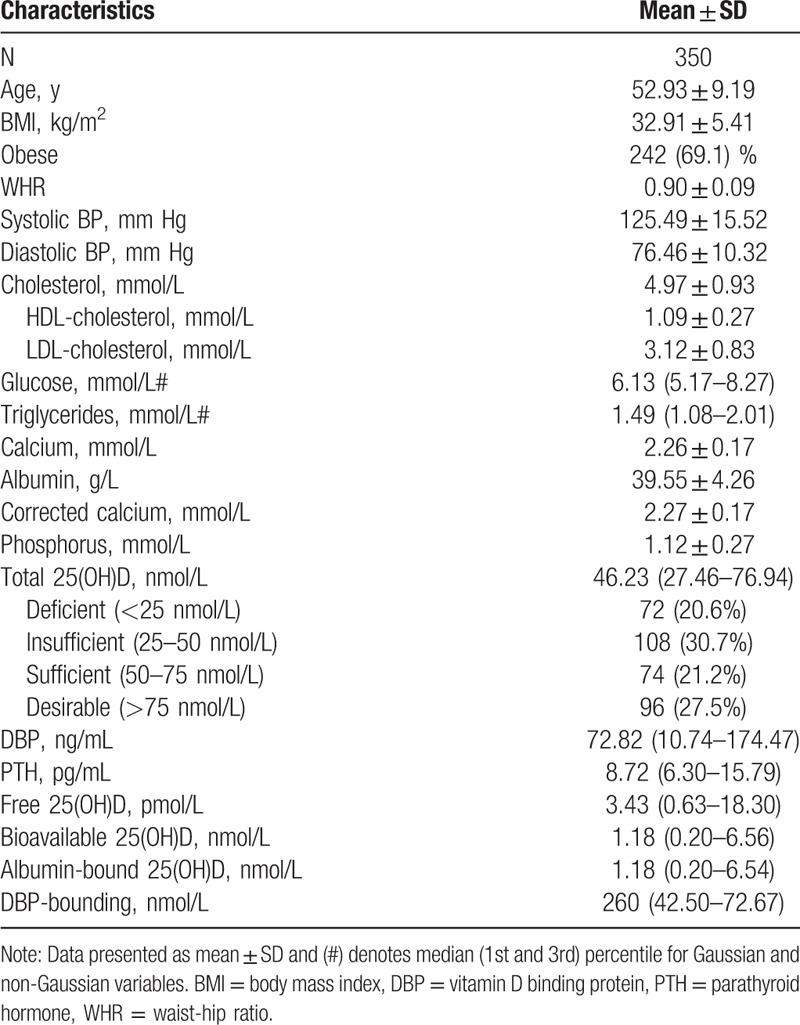
Characteristics of the study participants.

### 3.2 Associations between total, free, and bioavailable 25(OH)D and DBP

**Figure 1:**
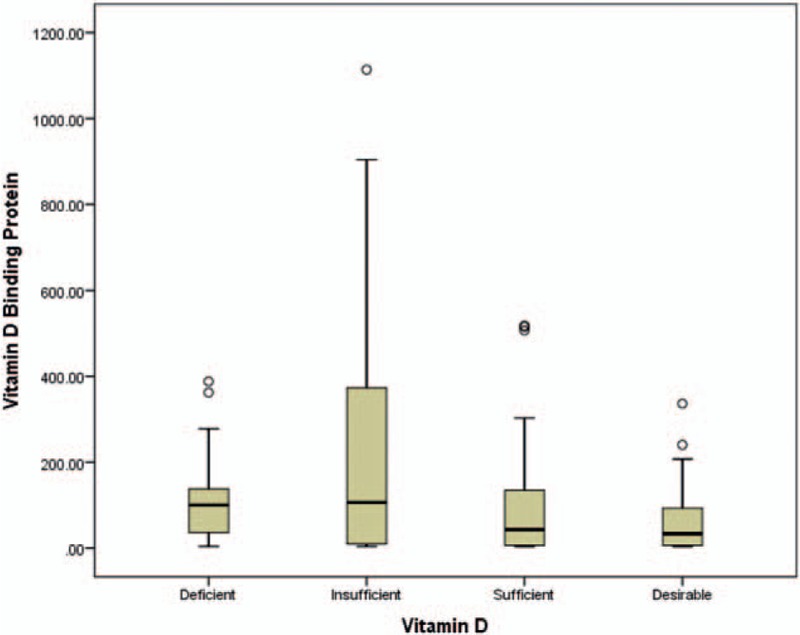
Boxplot of DBP levels by vitamin D status. DBP = vitamin D binding protein.

DBP was inversely and significantly associated with total 25(OH)D (r = −0.24; *P* < .01) as well as with free and bioavailable 25(OH)D (r = −0.9; *P* < .01) (Table 2). Differences between serum DBP levels and the 4 categories of 25(OH)D was found, whereas DBP at the lowest category was significantly different from that in other categories (*P* < .05 for all comparisons) (Fig. 1).

**Table 2:**
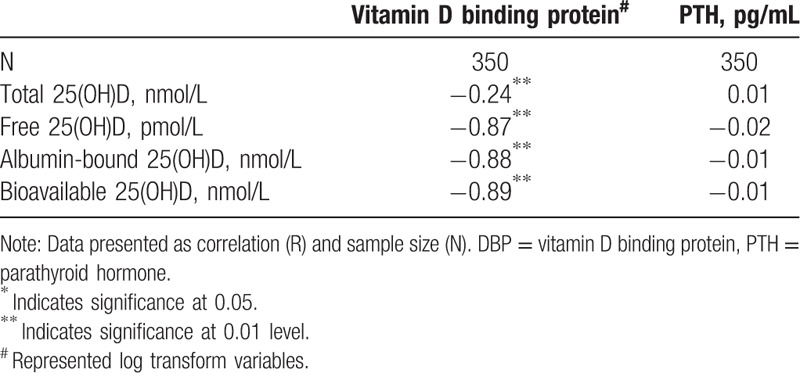
Correlation among vitamin D, DBP, and PTH with select variables.

**Figure 2:**
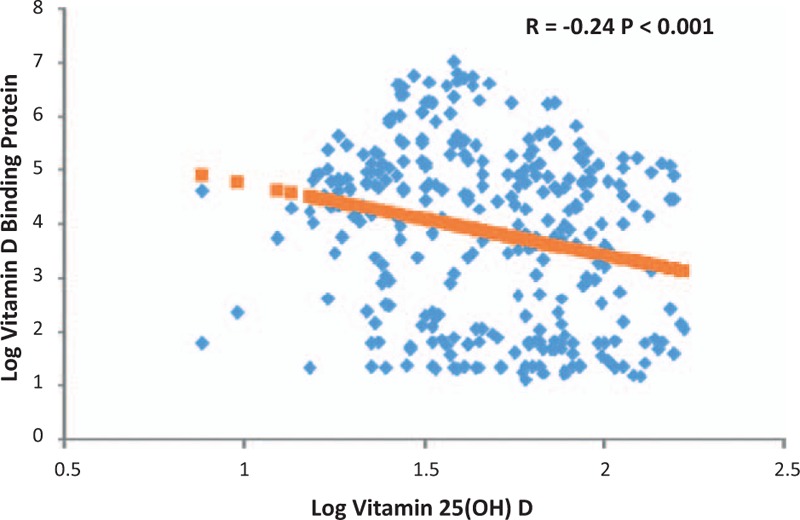
Relationship between DBP and vitamin D. DBP = vitamin D binding protein.

**Figure 3:**
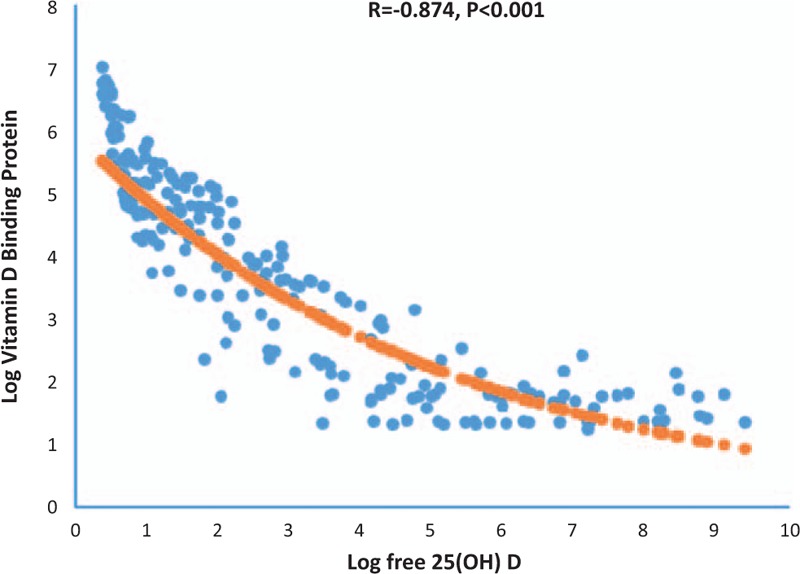
The association between percent free 25(OH)D with the level of DBP. DBP = vitamin D binding protein.

Linear regression model was found to give the best-fitting curve to describe the relationship between serum 25(OH)D levels and that of DBP. An inverse relationship was found between serum 25(OH)D and DBP (r = −0.24; *P* < .01) (Fig. 2). Non-linear plot of percent was observed between free 25(OH)D and DBP in (Fig. 3). At a DBP concentration above 300 Ag/mL, little further reduction in percent free 25(OH)D was observed.

### 3.3 Correlations between PTH and total, free, and bioavailable 25(OH)D

Without significance, a positive association was observed between PTH and total 25(OH)D, whereas an inverse association between PTH with free, and bioavailable 25(OH)D (Table 2). Further adjustment for DBP concentration did not alter the findings (data not shown). The associations among serum DBP, total, free, and bioavailable 25(OH)D and PTH level across quartiles of 25(OH)D were studied and only free vitamin showed significant inverse association in the 3rd quartile equivalent to sufficient vitamin D (Table 3).

**Table 3:**
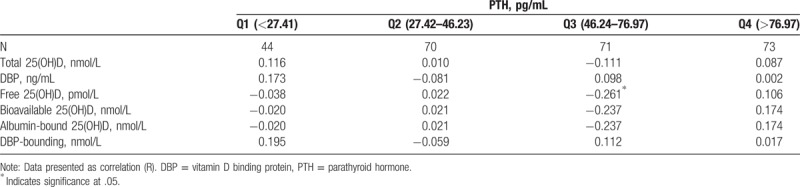
The associations among serum DBP, total, free, and bioavailable 25(OH)D, and PTH level across quartiles of 25(OH)D.

## 4 Discussion

To our knowledge, the present study is one of the few to examine the associations between total, free, and bioavailable 25(OH)D with PTH. Total 25(OH)D has been considered as the gold standard in measuring vitamin D status. Keeping in view that more than 99% of the circulating vitamin D is protein-bound, it is essential to evaluate if the biological effects of vitamin D is based on DBP levels or not. As only a small portion of circulating vitamin D and its metabolites can bind to albumin with low affinity compared to its affinity to DBP, the calculated free and bioavailable vitamin D provides a reasonable estimate of its bioactivity.^[10]^ In light of inconsistent reports, we evaluated relationship between free 25(OH)D, bioavailable 25(OH)D, total 25(OH)D, and DBP. Present study has found an extensive distribution of DBP among studied population and DBP also showed inverse associations with total 25(OH)D levels as well as with free and bioavailable 25(OH)D. It has been reported that the reduction in the concentration of DBP would release higher portion of bioavailable 25(OH)D accordingly.^[11]^ The inverse correlation between total, free, and bioavailable 25(OH)D and DBP suggests that synthesis of DBP may be a response to circulatory concentrations of total, free, and bioavailable 25(OH)D. These adaptive mechanisms of low DBP at high concentration of 25(OH)D, and bioavailable 25(OH)D may in fact represent vitamin D inadequacy.

DBP is synthesized and secreted primarily in the liver,^[12]^ a process that is affected by several factors (eg, estrogen and obesity).^[13]^ While estrogen levels per se are not normally elevated in obesity, higher levels of free estrogen in obesogenic states could influence hepatic DBP production.^[14]^ In a recent report, obese women had higher DBP concentrations and lower free 25(OH)D compared with normal-weight women.^[15]^ The serum DBP concentrations found in the obese women in our study (BMI = 32.91 ± 5.41) were not explained by a linear association with fat mass, and other factors not measured in this study may be involved in the higher concentrations of DBP. In vitro, IL-6 has been shown to increase hepatic DBP production,^[16]^ and IL-6 is raised in obesity and may also play a role in relation to the higher concentrations of DBP in the obese women in our study.^[17]^ Also, it has been reported that the DBP gene is moderately expressed in rat adipose tissue.^[18]^

Fluctuations in DBP can influence the dynamic equilibrium that exists between bound and free hormone fractions as well as govern the delivery of hormone to the target tissues. Thus, high DBP may serve as early indicator of perturbed vitamin D homeostasis. However, it has to be kept in mind that similar to many other circulating hormones, the free and bioavailable concentrations of 25(OH)D may be rigidly regulated by several mechanisms including PTH, phosphate, and many other factors. This suggests that the DBP does not impact the relationship between 25(OH)D and PTH. It was previously reported that there is lack of relationship between 25(OH)D and PTH in a cohort of vitamin D deficient nondiabetic and type 2 diabetic Saudi adults.^[19,20]^ No such evidence has been provided that shows the strength of free and/or bioavailable 25(OH)D and total 25(OH)D association with PTH. A study on hemodialysis patients observed that the strength of the association of free 25(OH)D with PTH is stronger than that of the associations of total 25(OH)D.^[21]^ Nevertheless, the dialysis subjects had significantly higher PTH and low albumin levels than healthy controls in our study.

Earlier studies have identified the interaction between 25(OH) D and DBP in pancreatic cancer diseases.^[22]^ Though our findings do not support that DBP directly influences the association between 25(OH)D and PTH, we did not find any direct influence of DBP on disease outcomes.

By the subdivision of vitamin D to quartiles, our results propose that bioavailable 25(OH)D is best parameter to measure the function of vitamin D than the total 25(OH)D, as a minimum in relation to vitamin D sufficiency. Moreover, use of bioavailable 25(OH)D could be better investigated to clarify the nature of the association between vitamin D and many diseases.^[9]^

This study has important strengths. It investigated the relationship between serum total, free, and bioavailable 25(OH)D versus PTH in racially homogenous groups of apparently healthy Saudi female adults considering the role of DBP which is a growing and intensely pursued field. Our findings substantiate the importance of considering free vitamin D forms in relevant clinical studies. The most important limitation of this study is the shortage of data on DBP polymorphisms and the cross-sectional design. Another limitation is the low sample size of the subgroups in the analysis across quartiles of 25(OH)D levels.

## 5 Conclusion

The present findings indicate that the biologically active 25(OH)D assessed by serum PTH is independent of DBP in healthy adults. DBP may not be that valuable in epidemiological studies measuring the associations between 25(OH)D and diseases that do not affect the DBP metabolism directly. Although, less availability of the literature on the cross-sectional design of the study and low sample size of the sub groups for the analysis across quartiles of 25(OH)D was not enough for a generalized conclusion.

## Acknowledgments

The authors also would like to thank the volunteers and the research team from the different primary care centers for the recruitment of subjects.
